# Relationship between tumour endothelial cell apoptosis and tumour blood flow shutdown following treatment with the antivascular agent DMXAA in mice

**DOI:** 10.1038/sj.bjc.6601606

**Published:** 2004-02-17

**Authors:** L-M Ching, S Zwain, B C Baguley

**Affiliations:** 1 Auckland Cancer Society Research Centre, Faculty of Medical and Health Sciences, The University of Auckland, Private Bag 92019, Auckland, New Zealand

**Keywords:** antivascular, blood flow, Hoechst 33342, knockout mice, tumour necrosis factor, apoptosis

## Abstract

5,6-Dimethylxanthenone-4-acetic acid (DMXAA) is currently undergoing clinical evaluation as an antivascular agent for the treatment of cancer. We have previously demonstrated that DMXAA induces apoptosis of vascular endothelial cells in murine tumour sections and in a breast carcinoma biopsy from one patient in a Phase I trial. We wished to determine the tissue selectivity of this effect and its relationship to induced blood flow changes. Mice with Colon 38 tumours were treated with DMXAA and tissues were examined for apoptosis by TdT-mediated dUTP nick-end labelling (TUNEL). Hoechst 33342 was used to stain functional vessels, with the loss of stained vessels used as a measure of tumour vascular collapse. Treatment with DMXAA at 25 mg kg^−1^, its maximum tolerated dose (MTD), showed, after 3 h, a 12-fold increase in TUNEL staining of tumour vascular endothelial cells. In contrast, tissue from the heart, brain, liver and spleen showed no increase. Induction of apoptosis in tumour tissue was both dose-dependent, observable at doses as low as 5 mg kg^−1^, and time-dependent. Apoptosis was significantly lower in Colon 38 tumours of mice, with a targeted disruption in the TNF gene (TNF^−/−^), or in the TNF receptor 1 gene (TNFR^−/−^), as compared with that in wild-type mice. Increasing the DMXAA dose to 50 mg kg^−1^ in these knockout mice raised tumour apoptosis to a level comparable to that induced in wild-type mice given DMXAA at the MTD. For all the data, a significant correlation (*r*=0.94; *P*<0.001) was found between logarithmic percentage apoptosis induction and the logarithmic density of Hoechst-stained vessels. These results suggest that blood flow inhibition caused by DMXAA is tumour tissue-specific and is a consequence of induction of apoptosis in tumour vascular endothelial cells.

5,6-dimethylxanthenone-4-acetic acid (DMXAA), a new anticancer agent synthesised in this laboratory (Rewcastle *et al*, 1991), is currently undergoing clinical evaluation as an antivascular agent for the treatment of cancer. In mice with transplantable tumours, DMXAA caused cessation of tumour blood flow, vascular collapse and tumour necrosis (Rewcastle *et al*, 1991; Zwi *et al*, 1994; Lash *et al*, 1998). DMXAA also increased tumour necrosis factor (TNF) concentrations in both plasma and tumour tissue of mice (Philpott *et al*, 1995; Ching *et al*, 1999). We have previously used TdT-mediated dUTP nick-end labelling (TUNEL) assays to demonstrate the induction of apoptosis of the vascular endothelium in Colon 38 tumours in mice treated with DMXAA at its optimal dose (Ching *et al*, 2002). Staining was detectable within 30 min of administration, intensified with time, and necrosis of adjacent tumour tissue was evident after 3 h. Some apoptosis of splenic tissue was detected in tumour-bearing mice, but none was observed in the liver tissue. Of particular interest was the finding of TUNEL staining of tumour vascular endothelium in breast tumour biopsies taken from a patient 3 and 24 h after infusion of DMXAA (3100 mg m^−2^) in a Phase I clinical trial. Thus, DMXAA is capable of inducing apoptosis in vascular endothelial cells in both mice and human tumours.

The finding of a rapid onset of tumour endothelial apoptosis, occurring before the appearance of detectable TNF in tumour tissue (Ching *et al*, 1999), suggests that DMXAA exerts a direct effect on tumour vasculature, and is of particular relevance to clinical trials. In this report, we have used *in vivo* vascular labelling techniques to investigate the relationship between apoptosis induction and tumour blood flow reduction. To investigate the role of TNF, we utilised mice with a targeted disruption of the TNF gene (TNF^−/−^) or of the TNF receptor 1 gene (TNFR^−/−^).

## MATERIALS AND METHODS

### Materials

DMXAA was synthesised at the Auckland Cancer Society Research Centre (Rewcastle *et al*, 1991) and dissolved in minimal 5% sodium bicarbonate for intraperitoneal injection into mice (25 mg kg^−1^) in a volume of 0.01 ml g^−1^ body weight. Hoechst 33342 (Sigma Chemical Co., St Louis, MO, USA) was dissolved at 8 mg ml^−1^ in saline and stored at −80°C.

### Mice

All mice were housed and used under institutional, ethical guidelines. All animal experiments have been carried out with ethical committee approval. The ethical guidelines that were followed meet the standards required by the UKCCCR guidelines (Workman *et al*, 1998). C57Bl/6 mice were obtained from the Animal Resource Unit, University of Auckland. TNF^−/−^ and TNFR^−/−^ knockout mice on a C57Bl/6 background were offspring from breeding pairs obtained, respectively, from the Centenary Institute, Sydney, Australia, and Jackson Laboratory, Bar Harbor, ME, USA. Colon 38 tumour fragments (1 mm^3^) were implanted subcutaneously in the left flank of anaesthetised (82 mg kg^−1^ sodium pentobarbitone) mice. Tumours were used when they had reached approximately 6 mm in diameter, generally 10 days after implantation. At least three mice were assigned for each group.

### Histochemistry

Apoptosis was determined using the TUNEL assay for the identification of double-stranded DNA breaks using the *In situ* Cell Death Detection Kit (Roche Diagnostics, Mannheim, Germany), according to the manufacturer's instructions. Tissue cryosections (14 *μ*m thickness) on poly-L-lysine-coated slides were fixed in 4% paraformaldehyde in phosphate-buffered saline (PBS) for 30 min at room temperature, washed three times with PBS for 10 min each time, dehydrated for 2 min in absolute ethanol and then treated with permeabilisation solution (1% Triton X-100 in 1% sodium citrate) for 15 min at room temperature. Strand breaks were labelled with fluoresceinated dUTP and visualised following reaction with either antifluorescein antibody conjugated with alkaline phosphatase and Vector® Black alkaline phosphatase substrate solution (Vector Laboratories, Burlingame, CA, USA) or antifluorescein antibody conjugated with horseradish peroxidase (POD) and diaminobenzidine (DAB) substrate (Roche Diagnostics, Mannheim, Germany). All slides were counter stained using methyl green. The amount of apoptotic staining in the sections was quantitated using Adobe Photoshop, Version 4 (Adobe Systems Inc., San Jose, CA, USA). For each of 5–10 random fields of tumour sections (2–3 tumours per group), the number of pixels stained with TUNEL was determined, divided by the total number of pixels, and expressed as a percentage.

Tissue cryosections were also fixed in cold acetone for 20 min at 4°C, blocked with 1.5% normal rabbit serum for 1 h at room temperature, incubated with avidin–biotin for 15 min, and then incubated with 1 : 100 dilution of rat anti-mouse CD-31 monoclonal antibody (MEC 13.3; BD Pharmingen, USA) overnight at 4°C in a humidified container. Sections were then incubated with 1 : 100 dilution of biotinylated anti-rat IgG antibody and avidin–biotin complex (Vectastain ABC-AP Kit, Vector Laboratories, Burlingame, CA, USA). Immunoglobulin complexes were visualised using Vector Red alkaline phosphatase substrate solution, also from Vector Laboratories.

### Hoechst 33342 staining of functional vessels

Hoechst 33342 (8 mg ml^−1^ in saline) was injected via the tail vein at 0.1 ml per mouse 3 h after DMXAA treatment. Mice were killed 2 min later by cervical dislocation and the tumours were excised and frozen at −80°C. Cryosections (14 *μ*m) of the tumour were examined using a fluorescence microscope with a UV-1A filter block (excitation 365 nm, barrier filter 400 nm, dichroic mirror 400 nm). Five–10 fields per tumour were scored (two to three tumours per group), and the number of positively stained vessels per 1 mm^−2^ field was calculated.

### Statistical analyses

Data were analysed using a paired Student's *t*-test and by standard correlation analysis. A probability value of <0.05 was considered significant.

## RESULTS

### Endothelial cell apoptosis in various tissues following DMXAA

**Figure 1:**
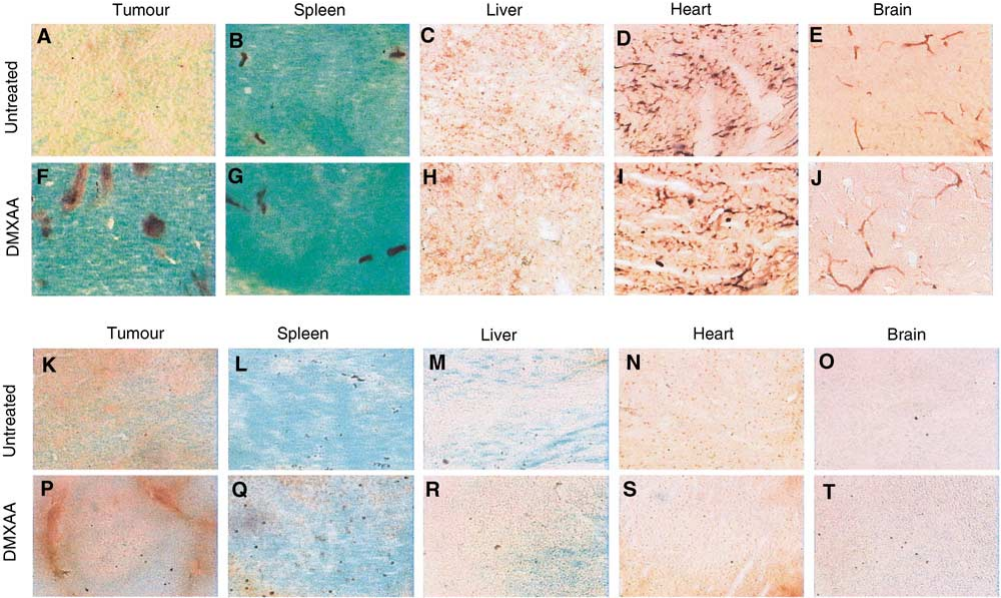
Selective induction of tumour vascular endothelial cell apoptosis by DMXAA. Sections from Colon 38 tumours, spleen, liver, heart and brain from untreated or treated (DMXAA, 25 mg kg^−1^, 3 h) C57Bl/6 mice were stained for TUNEL with alkaline phosphatase substrate (**A**–**J**) or POD/DAB (**K**–**T**). Stained sections shown at × 100 magnification.

**Figure 2:**
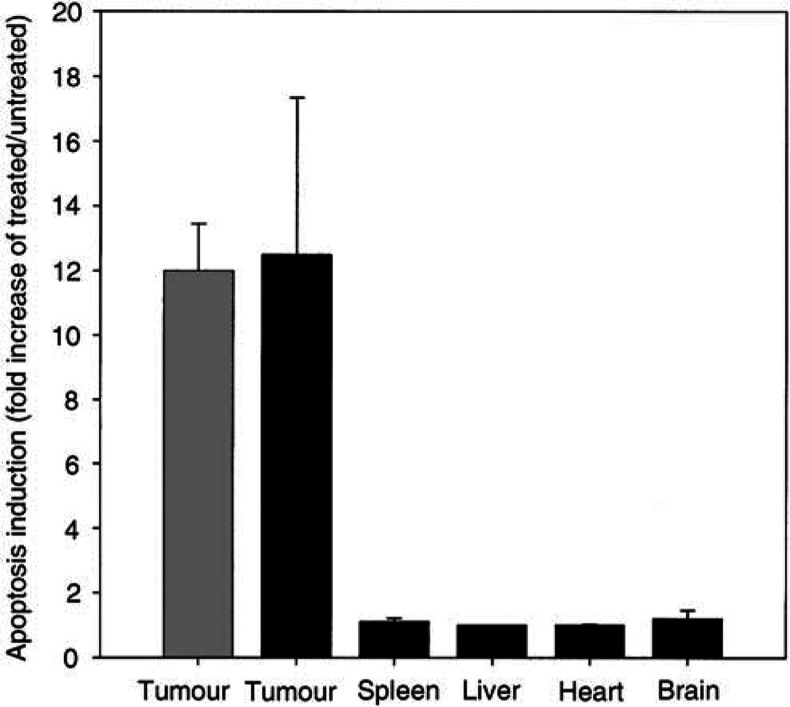
Apoptosis after 3 h in Colon 38 tumours, spleen, liver, heart and brain from mice treated with 25 mg kg^−1^ DMXAA. Bars represent ratios of percentage TUNEL-stained areas in the treated tissue to that in untreated tissue. Alkaline phosphatase substrate (black bars); POD/DAB substrate (grey bars).

Sections of Colon 38 tumours, liver, spleen, heart and brain collected from C57Bl/6 mice without treatment or 3 h after DMXAA administration (25 mg kg^−1^) were stained for apoptosis using TUNEL (Figure 1

). Tumour sections from DMXAA-treated mice showed 12.5- and 12-fold increases in apoptosis staining over that in tumour sections from untreated mice using alkaline phosphatase, or POD/DAB, respectively, as the enzyme system for visualisation of apoptosis staining (Figure 2

). No statistically significant increases in apoptosis staining were observed in other tissues (Figure 2). Liver sections from treated or untreated mice showed no staining. Using alkaline phosphatase, false-positive background staining was observed in the spleen, heart and brain sections of tumour-bearing and nontumour-bearing mice, and in negative control sections that had not been incubated with the immunohistochemistry reagents. Staining of sections of brain, liver, heart or spleen from treated or untreated mouse was not observed using the DAB substrate system, which confirmed that induction of apoptosis following DMXAA treatment was specific to tumour tissue (Figure 1). Similar ratios of apoptosis induction in tumour tissues were obtained using either POD/DAB or alkaline phosphatase (Figure 2), but the latter produced more intense labelling and was used in subsequent studies with tumour tissues.

To ascertain whether the apoptotic cells were endothelial cells, adjacent cryosections were stained with antibodies to CD-31 and the pattern of staining with the anti-CD-31 and apoptosis compared. Similar staining patterns were observed, providing strong evidence for DMXAA-induced endothelial apoptosis.

### Dose–response relationship and time course of DMXAA-induced tumour endothelial cell apoptosis and blood flow inhibition

A significant increase in apoptotic vessels in Colon 38 tumour sections, analysed 3 h after DMXAA treatment, was seen at doses as low as 5 mg kg^−1^ (Table 1

**Table 1:** Apoptosis induction and blood flow inhibition in Colon 38 tumours following DMXAA treatment

**Treatment**		**Endothelial cell apoptosis**		**Blood flow inhibition**	
**DMXAA (mg kg^−1^)**	**Time (h)**	**TUNEL stain (% area)**	**Increase over untreated**	**Hoechst-stained vessels mm^−2^**	**Percentage inhibition**
0	0	0.2±0.1		29.9±1.4	
5	3	0.5±0.1	2.5 (0.003)	28.8±1.2	4 (0.6)
10	3	0.8±0.3	4.0 (0.005)	26.2±1.3	13 (0.9)
15	3	1.0±0.2	5.0 (<0.001)	13.4±1.0	56 (<0.001)
20	3	1.2±0.1	6.0 (<0.001)	10.2±1.0	66 (<0.001)
25	3	2.5±0.9	12.5 (0.003)	7.2±0.7	76 (<0.001)
25	1	1.2±0.1	6.0 (<0.001)	12.5±0.6	58 (<0.001)
25	0.5	0.6±0.2	3.0 (<0.001)	18.4±1.2	39 (<0.001)
25	0.25	0.5±0.2	2.5 (0.003)	25.8±1.5	14 (0.08)

DMXAA=5,6-dimethylxanthenone-4-acetic acid. *P*-values in brackets represent the degree of statistical difference between treated and untreated controls.

, Figure 3A

**Figure 3:**
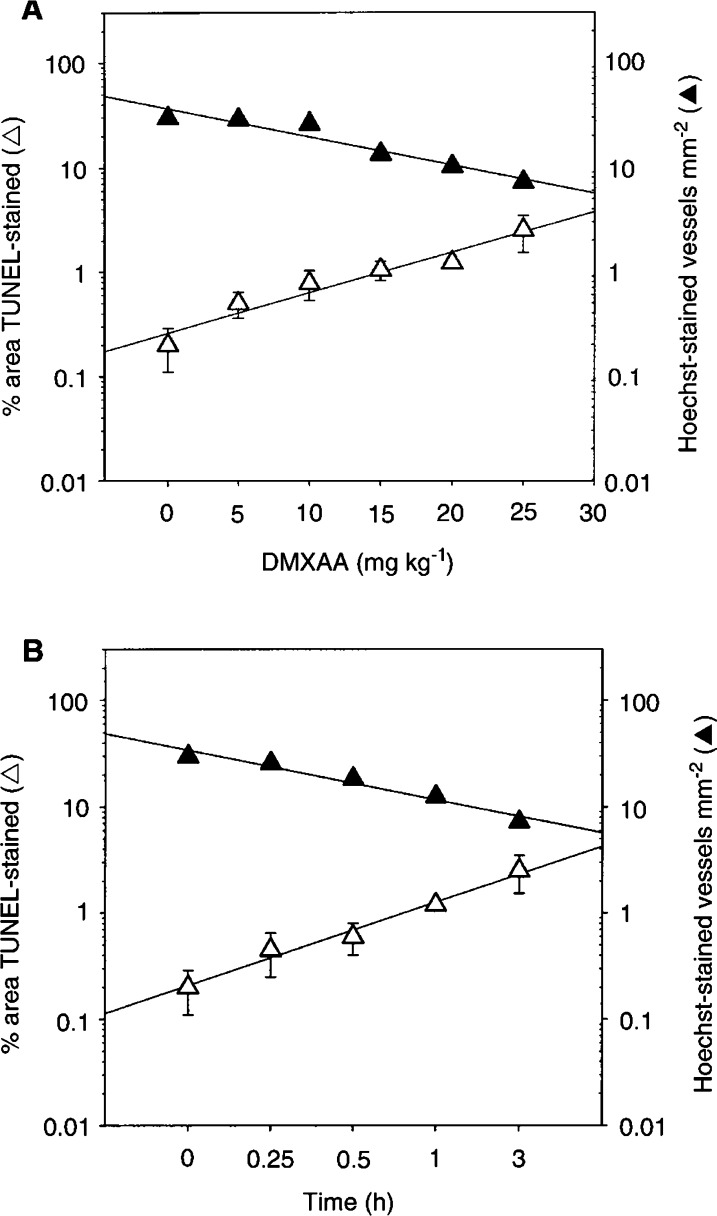
Apoptosis and blood flow measured in Colon 38 tumours treatment with DMXAA at different doses after 3 h (**A**), or at different times after DMXAA at a dose of 25 mg kg^−1^ (**B**). Percentage TUNEL-stained areas (Δ); Hoechst-stained vessels mm^−2^ (▴).

). The frequency of apoptotic vessels increased with increasing dose, with a particularly sharp increase from 20 mg kg^−1^ (six-fold induction as compared to untreated controls) to the MTD of 25 mg kg^−1^ (12.5-fold induction; Table 1). Apoptosis of tumour vascular endothelial cells was detectable as early as 15 min (2.5-fold increase) and progressively increased with time following administration of DMXAA at the MTD (Table 1, Figure 3B).

As a measure of blood flow inhibition following DMXAA treatment, we used the perfusion marker Hoechst 33342 to stain functional vessels (Zwi *et al*, 1989). No inhibition of blood flow was observed after 3 h with DMXAA doses of 5 and 10 mg kg^−1^. Inhibition was 56% at a dose of 15 mg kg^−1^ and increased progressively with dose up to the MTD (Table 1, Figure 3A). Blood flow was significantly reduced (39%) 30 min after DMXAA treatment at 25 mg kg^−1^, and reached 76% inhibition after 3 h (Table 1, Figure 3B).

### DMXAA-induced tumour endothelial cell apoptosis and blood flow shutdown in TNF^−/−^ and TNFR^−/−^ mice

To determine if the antivascular effects of DMXAA were TNF-dependent, we compared the responses in TNF^−/−^ and TNFR^−/−^ mice to those in wild-type C57Bl/6 mice. Tumour endothelial cell apoptosis in TNF^−/−^ and TNFR^−/−^ hosts following DMXAA (25 mg kg^−1^) was, respectively, 1.8- and 10.4-fold lower than that in wild-type mice. However, the knockout mice tolerated higher doses of DMXAA and, at a dose of 50 mg kg^−1^, the induced apoptosis was comparable to that obtained in wild-type mice at 25 mg kg^−1^ of DMXAA in wild-type mice. Blood flow in tumours implanted in TNF^−/−^ and TNFR^−/−^ mice was determined from Hoechst-stained vessels, and was, respectively, 2.5- and 5.3-fold lower than that in tumours in wild-type mice, 3 h following DMXAA at 25 mg kg^−1^. Again, however, at the higher dose of 50 mg kg^−1^, which can be tolerated by the knockout mice, inhibition of blood flow was similar to that obtained at 25 mg kg^−1^ in wild-type mice (Table 2

**Table 2:** Endothelial cell apoptosis and blood flow inhibition in tumours from C57Bl/6, TNF^–/−^ and TNFR^–/−^ mice following DMXAA treatment

	**Percentage TUNEL-stained areas**			**Hoechst-stained vessels mm^−2^**		
**DMXAA**	**C57Bl/6**	**TNF^−/−^**	**TNFR^−/−^**	**C57Bl/6**	**TNF^−/−^**	**TNFR^−/−^**
0	0.2±0.1	0.2±0.1	0.3±0.1	29.9±1.4	32.5±1.7	35.7±2.7
25 mg kg^−1^	2.5±1.0	1.4±0.5	0.3±0.1	5.6±1.0	13.6±1.4	31.3±1.3
50 mg kg^−1^	—	2.8±0.8	1.9±0.6	—	6.0±0.5	6.0±0.6

DMXAA=5,6-dimethylxanthenone-4-acetic acid.

).

## DISCUSSION

These results confirm our previous findings (Ching *et al*, 2002) that DMXAA induces endothelial cell apoptosis in Colon 38 tumours. Apoptosis induction was selective to tumour vascular endothelium and was not seen in liver, heart, brain or spleen (Figures 1 and 2). We had previously reported apoptosis staining in splenic tissues, using alkaline phosphatase for the detection of the bound antibodies (Ching *et al*, 2002), but the results here show that the staining observed in the normal organs using the alkaline phosphatase procedure was not DMXAA-induced and was likely to be due to high endogenous phosphatase levels that had not been completely blocked (Figure 2). The basis for the pronounced selectivity for tumour vasculature is not yet understood. Factors secreted by tumour-associated immune cells, or by the tumour cells themselves, may play a role by ‘priming’ the response of tumour endothelial cells to DMXAA. Tumour-conditioned medium has been reported to play a role in modulating the response of cultured endothelial cells to flavone acetic acid (Watts and Woodcock, 1992). Endothelial cells in culture are resistant to apoptosis induction by DMXAA (Ching *et al*, 2002), and we have found that addition of serum from Colon 38-bearing mice did not render them sensitive (unpublished results).

**Figure 4:**
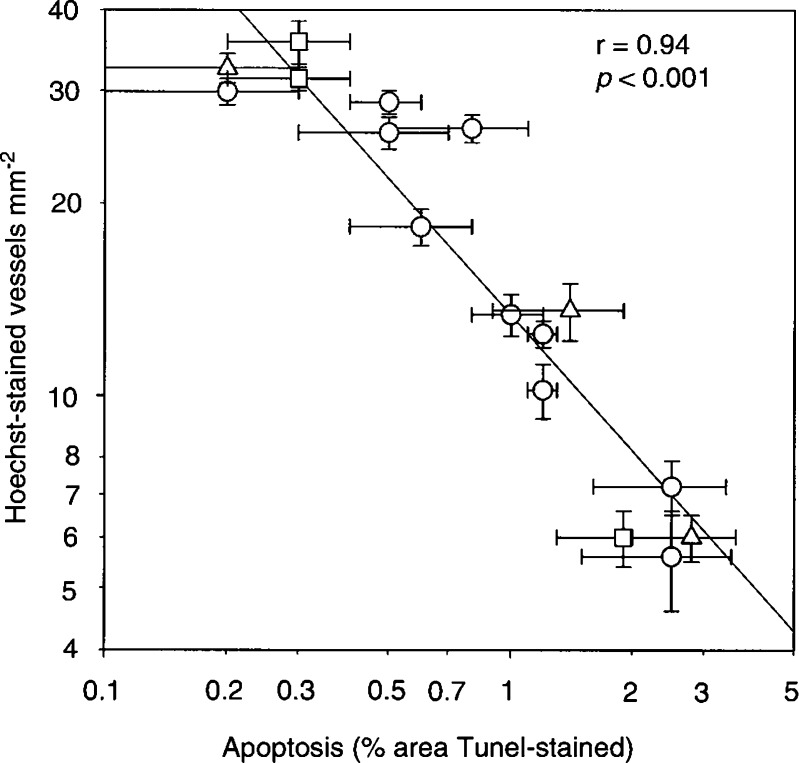
Relationship between the logarithm of induced apoptosis and the logarithm of blood flow inhibition in Colon 38 tumours, plotted for all the experiments. Wild-type C57Bl/6 (○); TNF^−/−^ (Δ); TNFR^−/−^ (□).

To determine whether there was a relationship between the degree of blood flow inhibition and endothelial cell apoptosis induction, all the data for both wild-type and knockout mice treated with DMXAA with different doses and at different times were plotted on the same graph (Figure 4

). A highly significant logarithmic relationship was found (*r*=0.94; *P*<0.001), indicating that a 10% increase in apoptosis leads to a 7% decrease in blood flow. The degree of significance suggests that tumour blood flow inhibition is a consequence of endothelial cell apoptosis. Damage to the endothelium and subsequent loss of the structural integrity of the vessels leading to increase in vascular permeability would result in a reduction in blood flow (Baguley, 2003).

TNF is induced following DMXAA administration to mice (Philpott *et al*, 1995), and the histology of tumours treated with DMXAA resembles that of TNF-treated tumours, suggesting that TNF participates in the antivascular action. Support for this hypothesis is provided by experiments where Colon 38 tumours were implanted in TNF^−/−^ and TNFR^−/−^ knockout mice, where the antitumour effects following administration of the same dose of DMXAA are substantially reduced (Ching *et al*, 1999; Zhao *et al*, 2002). In agreement with these findings, apoptosis induction and tumour blood flow inhibition following treatment with DMXAA (25 mg kg^−1^) were pronounced in tumours implanted in wild-type mice, but small in tumours implanted in TNF^−/−^ and TNFR^−/−^ knockout mice (Table 2). The lower toxicity of DMXAA in these knockout mice allows the use of higher drug doses, which restored both apoptosis induction and tumour blood flow inhibition responses. The results are consistent with the hypothesis that DMXAA can exert an antivascular response both directly and indirectly by induction of TNF, and perhaps of other cytokines. The relationship in Figure 4 suggests that both direct and indirect mechanisms act with a similar relationship between apoptosis induction and tumour blood flow inhibition. These results are of particular importance to clinical studies, since TNF levels were not found to be raised in Phase I clinical trials of DMXAA but tumour blood flow shutdown at doses above 500 mg m^−2^ was clearly demonstrable (Rustin *et al*, 1998; Jameson *et al*, 2003). Multiple mediators of antivascular effects may be involved in providing a selective antitumour effect.
